# Infectious SARS-CoV-2 in Feces of Patient with Severe COVID-19

**DOI:** 10.3201/eid2608.200681

**Published:** 2020-08

**Authors:** Fei Xiao, Jing Sun, Yonghao Xu, Fang Li, Xiaofang Huang, Heying Li, Jingxian Zhao, Jicheng Huang, Jincun Zhao

**Affiliations:** Sun Yat-sen University, Zhuhai, China (F. Xiao);; Guangzhou Medical University, Guangzhou, China (J. Sun, Y. Xu, F. Li, X. Huang, Jingxian Zhao, Jincun Zhao);; Chinese Academy of Sciences, Guangzhou (H. Li);; Guangzhou Customs District Technology Center, Guangzhou (J. Huang)

**Keywords:** severe acute respiratory syndrome coronavirus 2, SARS-CoV-2, viruses, coronavirus, coronavirus disease, COVID-19, respiratory infections, virus isolation, feces, transmission, zoonoses

## Abstract

Severe acute respiratory syndrome coronavirus 2 was isolated from feces of a patient in China with coronavirus disease who died. Confirmation of infectious virus in feces affirms the potential for fecal–oral or fecal–respiratory transmission and warrants further study.

Severe acute respiratory syndrome coronavirus 2 (SARS-CoV-2) recently emerged in China, causing a major outbreak of severe pneumonia and spreading to >200 other countries (*1*). As of May 5, 2020, a total of 3,517,345 cases of coronavirus disease (COVID-2019) and 243,401 deaths had been reported to the World Health Organization (https://www.who.int/docs/default-source/coronaviruse/situation-reports/20200505covid-19-sitrep-106.pdf?sfvrsn=47090f63_2). The virus is believed to be spread by direct contact, fomites, respiratory droplets, and possibly aerosols (*2*). Viral RNA has been detected in feces and urine of some patients (*3**–**7*). Infectious virus was also isolated from urine of a patient with severe COVID-19 (*8*). However, it is unclear whether the virus in feces is infectious and might be an additional source for transmission. 

This study was approved by the Health Commission of Guangdong Province and the Ethics Committees of Guangzhou Medical University to use patient and healthy donor sample specimens. On January 17, 2020, a 78-year-old man who had a history of recent travel to Wuhan, China, was admitted to the Fifth Affiliated Hospital of Sun Yat-Sen University because of a cough for 7 days and intermittent fever (Appendix Figure 1, panel A). Computed tomography of his chest showed multiple, ground-glass opacities (Appendix Figure 2). Nasopharyngeal and oropharyngeal swab specimens were positive for SARS-CoV-2 RNA by quantitative reverse transcription PCR (qRT-PCR).

On January 22, the patient’s condition deteriorated and he was intubated. Ventilator-assisted breathing was instituted. The first feces specimen was collected on January 27 and was positive for viral RNA by qRT-PCR. Serial feces samples were collected on January 29, February 1, and February 7. All samples were positive for viral RNA (Appendix Figure 1, panel A). Viral antigen was also detected in gastrointestinal epithelial cells of a biopsy sample, as reported (*9*). The patient died on February 20.

We collected fecal specimens on January 29 to inoculate Vero E6 cells. Cycle threshold values for the fecal sample were 23.34 for the open reading frame 1lab gene and 20.82 for the nucleoprotein gene. A cytopathic effect was visible in Vero E cells 2 days after a second-round passage (Appendix Figure 1, panel B). We extracted viral nucleic acid from virus culture supernatant by using the QIAamp Viral RNA Extraction Kit (QIAGEN, https://www.qiagen.com) and obtained full-length viral genome sequence (GenBank accession no. MT123292) by using next-generation sequencing. The sequenced showed 5 nt substitutions compared with the original Wuhan strain (GenBank accession no. NC045512.2) (Appendix Table). 

We negatively stained culture supernatant and visualized by transmission electron microscopy. Viral particles that were visible were spherical and had distinct surface spike protein projections, consistent with a previously published SARS-CoV2 image (Appendix Figure, panel C) (*1*).

To estimate viral loads (log_10_ PFU equivalents/mL) in clinical samples from qRT-PCR cycle threshold values, we generated a standard curve from a serially diluted SARS-CoV-2 of known plaque titer. Viral loads quantified by using this method were viral RNA levels, not of infectious virus. The viral load was higher in feces than in respiratory specimens collected at multiple time points (17–28 days after symptom onset) (Appendix Figure, panel D). Isolation of virus from feces samples collected at later time points was not successful, although results for virus RNA remained positive, indicating only RNA fragments, not infectious virus, in feces of this patient collected at later time points of disease onset. 

We collected feces specimens from 28 patients; 12, including the patient described in this report, were positive for viral RNA for >1 time point. We attempted to isolate SARS-CoV-2 virus from 3 of the viral RNA–positive patients. Results were successful for 2 of 3 patients, including the patient from this report, indicating that infectious virus in feces is a common manifestation of COVID-19. 

The patient from this report had a high level of IgG against spike protein. Levels of nucleocapsid protein–specific antibodies were relatively lower. Spike protein (1,274 aa) is much larger than nucleoprotein protein (420 aa), which potentially contains more epitopes inducing specific antibody responses.

We also identified neutralization antibodies by using a focus reduction neutralization test. Neutralizing titers (50% focus reduction neutralization test) ranged from 1:1,065 to >1:4,860 at different time points (Appendix Figure, panel E). To show that isolated virus was infectious to susceptible cells, we tested fresh Vero E6 cells infected with the virus isolate by using indirect immunofluorescent assay and serum samples from the patient and a healthy donor. A positive reaction was only obtained with the patient serum (Appendix Figure 1, panel F).

Isolation of infectious SARS-CoV-2 in feces indicates the possibility of fecal–oral transmission or fecal–respiratory transmission through aerosolized feces. During the 2003 severe acute respiratory syndrome pandemic, 329 residents of a private housing estate in Hong Kong were infected; 42 died (*10*). Investigation of the building’s structure showed that faulty sewage pipelines led to aerosolization of contaminated feces, which was believed to be the source of infection. 

Our findings indicate the need for appropriate precautions to avoid potential transmission of SARS-CoV-2 from feces. Discharge and hospital cleaning practices should consider this possibility for critically ill patients or those who died who had high viral loads and are more likely to shed infectious virus.

Appendix Additional information on infectious SARS-CoV-2 in feces of patient with severe COVID-19.
