# Supplementary material for: Infectious SARS-CoV-2 in Feces of Patient with Severe COVID-19
Source: Emerg Infect Dis. 2020 Aug;26(8):1920–2. doi: 10.3201/eid2608.200681 (PMC7392466; doi:10.3201/eid2608.200681)
Supplement: Appendix — Additional information on infectious SARS-CoV-2 in feces of patient with severe COVID-19. [file 20-0681-Techapp-s1.pdf]

Article DOI: https://doi.org/10.3201/eid2608.200681

Infectious SARS-CoV-2 in Feces of Patient with Severe COVID-19

Appendix

Methods

Virus Isolation and Transmission Electron Microscopy

Vero E6 cells were used for virus isolation. Cells were cultured in Dulbecco modified Eagle medium (DMEM) containing 10% fetal bovine serum (FBS). A quantitative reverse transcription PCR (qRT-PCR)–positive fecal swab specimen was saved in viral transport media (DMEM containing 1% bovine serum albumin, 15 μg/mL amphotericin, 100 units/mL penicillin G, and 100 μg/mL streptomycin). Before virus isolation, the sample was filtered with 0.45-μm strainer and diluted 1:10 with DMEM containing 2% FBS and antimicrobial drugs. Cells were infected at 37°C for 1 h. The inoculum was removed and replaced with fresh culture medium. The cells were incubated at 37°C and observed daily for a cytopathic effect. If there was no obvious cytopathic effect until day 6 postinfection, the cells and supernatant were scraped up, freeze-thawed once, and overlaid to new cells for second-round passage. Culture supernatant was negatively stained and visualized by transmission electron microscopy.

qRT-PCR

Viral RNA from respiratory and fecal swab specimens was extracted by using the Nucleic Acid Isolation Kit (Zybio Inc., https://m.zybio.com). A real-time PCR assay kit targeting severe acute respiratory syndrome coronavirus 2 (SARS-CoV-2) open reading frame 11ab and nucleoprotein gene regions was provided by Zybio Inc. Serial sampling of nasopharyngeal swab, oropharyngeal swab, and fecal swab specimens were used to monitor viral shedding during SARS-CoV-2 infection. To estimate viral loads in clinical samples from qRT-PCR cycle threshold values, a standard curve was generated from a serially diluted SARS-CoV-2 standard of known plaque titer. Viral loads in clinical samples were then calculated as $\log_{10}$ PFU equivalents/mL.

Complete Genome Sequencing

Full-length viral genome sequence was obtained by using next-generation sequencing. Viral RNA was extracted from virus culture supernatant by using the QIAamp Viral RNA Extraction Kit (QIAGEN, https://www.qiagen.com) according to the manufacturer's instructions. The concentration of RNA was determined by using a Qubit 4 Fluorometer (ThermoFisher Scientific, https://www.thermofisher.com), and 50–1,000 ng RNA were mixed with rRNA deletion probes for rRNA depletion. Library construction was then completed by using the RNA library construction kit (Vision Medicals, http://m.visionmedicals.com), which including fragmentation, the synthesis of first and second strands, end repairment, and adaptor ligation. The amplified libraries of each sample were pooled and sequenced on an Illumina Nextseq Sequencer (https://www.illumina.com) for clinical metagenomic analysis. A minimum of 10 million single-end 75-bp reads were obtained per sample. Sequence analysis was performed by using the Vision Medicals IDseqTM commercial bioinformatic pipeline. In brief, low-quality and short (length < 35 bp) reads and reads that mapped to human genome and plasmids were removed. The remaining reads were taxonomically classified by aligning curated microbial database consisting of viruses, bacteria, fungi, and parasites. The taxonomic references were downloaded from National Center Biotechnology Information (Bethesda, MD, USA). Upon identification of critical pathogen, the identified species-specific sequences were further confirmed by using blastn (https://blast.ncbi.nlm.nih.gov) for further accuracy validation. Complete genome was aligned to SARS-CoV-2 reference genome NC045512.2.

SARS-CoV-2 Spike- and Nucleoprotein-Specific IgG ELISAs

Serum samples were collected and used to analyze SARS-CoV-2–specific IgG against SARS-CoV-2 spike and nucleocapsid proteins in ELISAs. Spike and nucleocapsid proteins were coated on ELISA plates at a concentration of 50 ng/well overnight at 4°C. After blocking in Dulbecco phosphate-buffered saline and 10% FBS, 100 μL diluted plasma (1:100) were added, and plates were incubated at 37°C for 1 h. After washing, plates were incubated with 100 μL of horseradish peroxidase–conjugated mouse anti-human IgG (heavy plus light chain) (Jackson ImmunoResearch, https://www.jacksonimmuno.com) at 37°C for 1 h. Reactions were visualized by adding 50 μL of 3,3',5,5'-tetramethylbenzidine substrate solution (Biohao Biotechnology Co., Ltd., http://www.biohao.com. Optical densities at 450 nm were then read. A serum sample from a healthy donor was used as a negative control, and a serum sample from a confirmed coronavirus disease patient was used as a positive control.

Focus Reduction Neutralization Test

A SARS-CoV-2 focus reduction neutralization test was performed in a certified Biosafety Level 3 Laboratory. Plasma samples (75 μL/sample) were serial diluted, mixed with 75 μL of SARS-CoV-2 (8×10^3 FFU/mL) in 96-well microwell plates, and incubated for at 37°C for 1 h. Mixtures were then transferred to 96-well plates seeded with Vero E6 cells and incubated (to enable absorption) at 37°C for 1 h. Inoculums were then removed before adding the overlay media (100 μL minimum Eagle medium containing 1.6% carboxymethylcellulose. The plates were then incubated at 37°C for 24 h. Cells were fixed with 4% paraformaldehyde solution for 30 min, and overlays were removed. Cells were permeabilized with 0.2% Triton X-100 and incubated with cross-reactive rabbit anti-SARS-CoV-N IgG (Sino Biological, Inc., https://www.sinobiological.com) for 1 h at room temperature before adding horseradish peroxidase–conjugated–conjugated goat anti-rabbit IgG (heavy plus light chain) (Jackson ImmunoResearch). Cells were further incubated at room temperature. The reactions were developed with KPL TrueBlue Peroxidase substrates (Sera care Life Sciences Inc., https://www.seracare.com). The numbers of SARS-CoV-2 foci were calculated by using an EliSpot reader (Cellular Technology Ltd.; http://www.immunospot.com).

Indirect Immunofluorescence Assay

An indirect immunofluorescence assay IFA was performed by using Vero E6 cells grown on cover slips that were infected with the virus isolate at a multiplicity of infection of 0.05 for 24 h. Viral antigens were detected by using patient serum as primary antibody and Alexa Fluor 488 dye–labeled goat anti-human IgG as secondary antibody. Nuclei were counterstained with 4′,6-diamidino-2-phenylindole. Serum from a healthy donor was used as a negative control. The slips were observed by using a laser scanning confocal microscope (LSM 880; Zeiss, https://www.zeiss.com).

Appendix Table. Nucleotide and amino acid changes between original coronavirus virus strain (Wuhan-Hu-1 NC_045512.2) and virus isolated from the feces of the patient

Genome nucleotide position	Protein	Nucleotide changes		Amino acid changes
		NC_045512.2	MT123292	
654	ORF1ab	G	A	Gly > Glu
6819	ORF1ab	G	T	Ser > ILe
6996	ORF1ab	T	C	Ile > Thr
17373	ORF1ab	C	T	Synonymous
29527	N	G	A	Synonymous

*N, nucleoprotein; ORF, open reading frame.

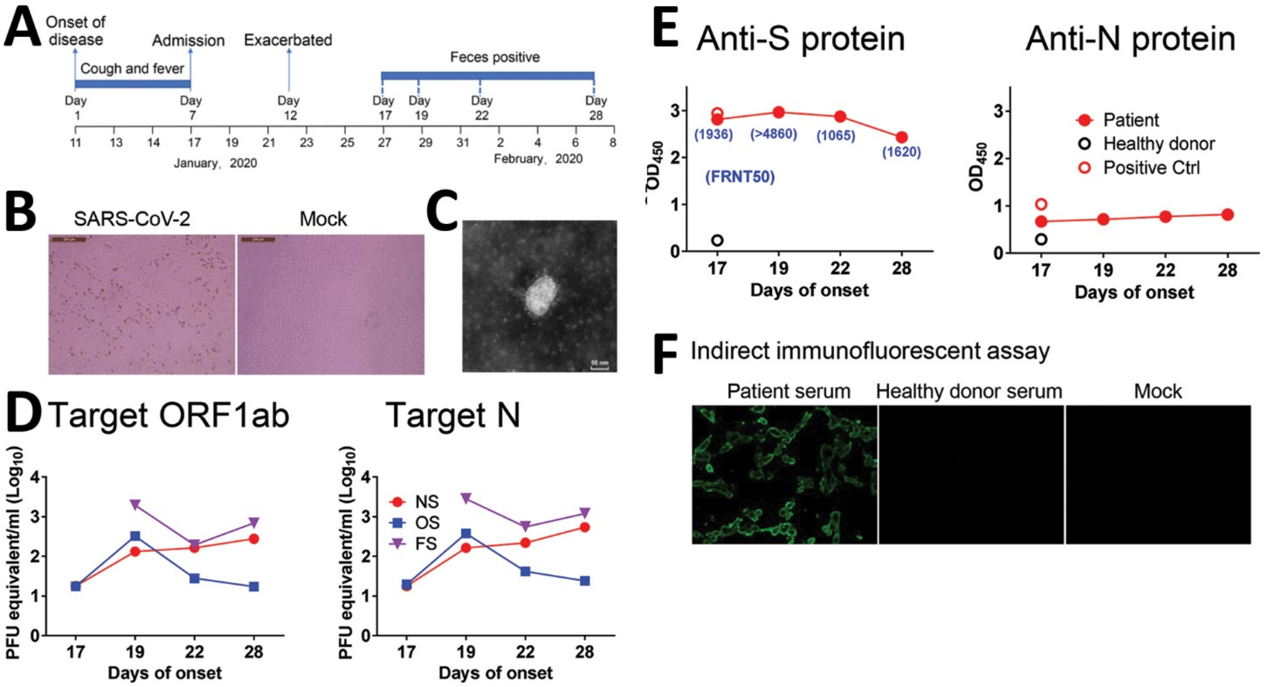

Appendix Figure 1. Clinical information and isolation of SARS-CoV-2 from feces of a patient. A) Clinical events. B) Vero E6 cells infected with SARS-CoV-2 isolate for 72 hours. C) Detection of viral particles by using transmission electron microscopy (original magnification, ×98,000). D) Viral loads in respiratory and fecal specimens. NS, nasopharyngeal swab specimen; OS, oropharyngeal swab specimen; FS, fecal swab specimen. E) SARS-CoV-2–specific antibody against spike (S) and nucleoprotein (N) in patient and neutralizing antibody. F) Indirect immunofluorescent assay detection of SARS-CoV-2–infected Vero E6 cells by using patient serum collected on February 7, 2020 (28 days postonset of illness) (original magnification ×200). Ctrl, control; FRNT50, 50% focus reduction neutralization test; ORF, open reading frame; SARS-CoV-2, severe acute respiratory syndrome coronavirus 2.

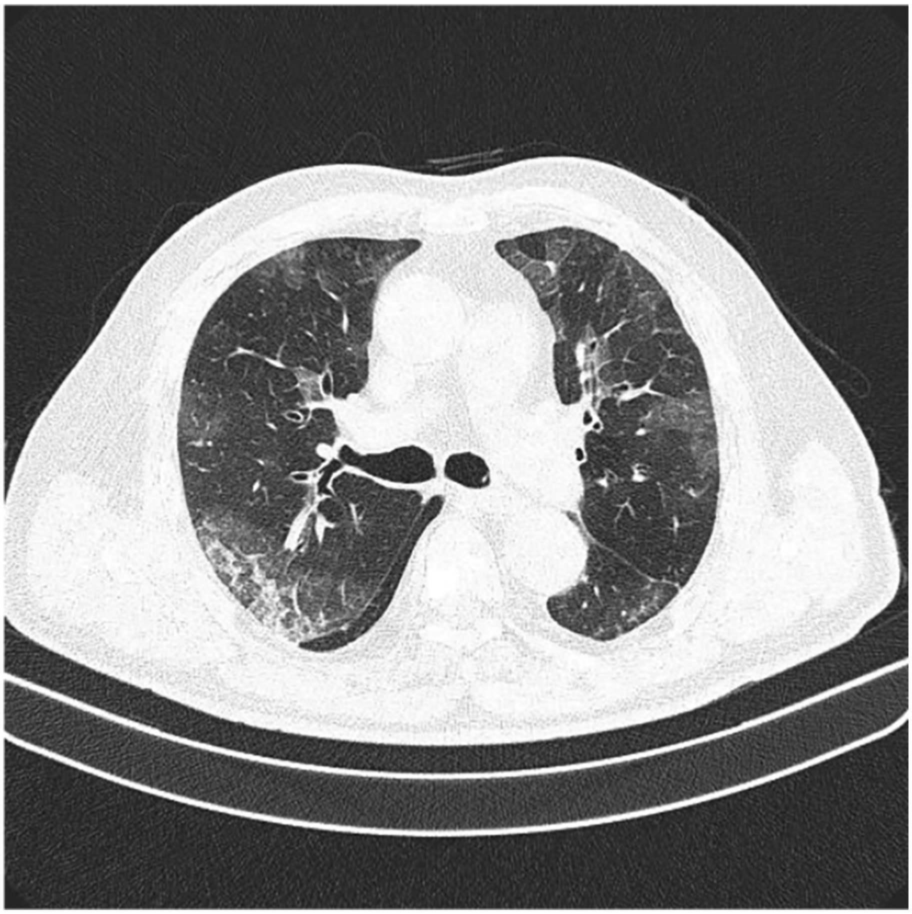

Appendix Figure 2. Chest computed tomography of the patient infected with severe acute respiratory syndrome coronavirus 2.
